# Audit of patients absent without leave from a psychiatric intensive care unit

**DOI:** 10.1192/bjo.2021.283

**Published:** 2021-06-18

**Authors:** Hamish Naismith, Mehtab Rahman, Biganani Magadlela

**Affiliations:** Central and North West London NHS Foundation Trust

## Abstract

**Aims:**

We aimed to reduce the number of patients absent without leave (AWOL) by carrying out an audit of processes around granting leave for those patients and the action taken when they absconded. We also wanted to determine factors which might be associated with patients absconding.

**Background:**

Nile ward is a 14-bedded male psychiatric intensive care unit (PICU). All patients admitted to the ward are under section 2 or 3 of the Mental Health Act. Patients who are AWOL may pose a risk of harm to themselves or others. The Royal College of Psychiatrists’ Quality Network for PICUs has developed applicable standards, which include criteria on developing a leave plan, actions to take when patients are AWOL and involvement of carers.

**Method:**

Patients who went AWOL during a six month period in 2019 from ward records. The electronic medical records for identified patients were reviewed to assess whether the following eight criteria were met: risk assessment documented; leave conditions specified; consultation with the multi-disciplinary team; crisis card provided to patients or families; risk management plan enacted when AWOL; relevant authorities informed; incident form completed; relatives/carers involved in patient's care if they consented. In reviewing the notes, factors that might have been associated with an increased risk of AWOL were also assessed in order to inform risk assessment.

**Result:**

Six patients were identified who went AWOL during the six month period in question. For six of the criteria, all of patients' cases met the audit standards. Five patients' did have involvement of relaties/carers if they consented, but in one case no details were available for making contact. All patients lacked documented details of crisis numbers being provided before they went on leave. Preliminary findings that might be associated with an increased risk of AWOL are differing views between the patient and the treating team on the care plan and concerns about mental state.

**Conclusion:**

The audit showed many of the standards are met. However, a quality improvement intervention is planned to ensure all audit standards are met, in particular around providing a crisis card to patients and these findings will be presented on the poster, if accepted. Further research is needed into factors which might be associated with an increased risk of absconsion in PICU.

